# The Program Sustainability Assessment Tool: A New Instrument for Public Health Programs

**DOI:** 10.5888/pcd11.130184

**Published:** 2014-01-23

**Authors:** Douglas A. Luke, Annaliese Calhoun, Christopher B. Robichaux, Michael B. Elliott, Sarah Moreland-Russell

**Affiliations:** Author Affiliations: Annaliese Calhoun, Christopher B. Robichaux, Sarah Moreland-Russell, Washington University in St Louis, Missouri; Michael B. Elliott, Saint Louis University, St Louis, Missouri.

## Abstract

**Introduction:**

Public health programs can deliver benefits only if they are able to sustain programs, policies, and activities over time. Although numerous sustainability frameworks and models have been developed, there are almost no assessment tools that have demonstrated reliability or validity or have been widely disseminated. We present the Program Sustainability Assessment Tool (PSAT), a new and reliable instrument for assessing the capacity for program sustainability of various public health and other programs.

**Methods:**

A measurement development study was conducted to assess the reliability of the PSAT. Program managers and staff (n = 592) representing 252 public health programs used the PSAT to rate the sustainability of their program. State and community-level programs participated, representing 4 types of chronic disease programs: tobacco control, diabetes, obesity prevention, and oral health.

**Results:**

The final version of the PSAT contains 40 items, spread across 8 sustainability domains, with 5 items per domain. Confirmatory factor analysis shows good fit of the data with the 8 sustainability domains. The subscales have excellent internal consistency; the average Cronbach’s α is 0.88, ranging from 0.79 to 0.92. Preliminary validation analyses suggest that PSAT scores are related to important program and organizational characteristics.

**Conclusion:**

The PSAT is a new and reliable assessment instrument that can be used to measure a public health program’s capacity for sustainability. The tool is designed to be used by researchers, evaluators, program managers, and staff for large and small public health programs.

## Introduction

The new discipline of dissemination and implementation science has driven an increase in studies of how new scientific discoveries are translated and developed into programs, policies, and practices (1). The evidence base in dissemination and implementation science is growing, especially in the health sciences (2). However, dissemination and implementation science has paid much less attention to what happens to programs once they have been implemented. Even those studies that focus on examining implementation of programs do so in terms of immediate or short-term implementation rather than long-term outcomes. Maintaining effective public health programs once they are implemented is often challenging, given rapid changes in budgetary and political climates. Public health programs can deliver benefits only if they are able to reach a certain level of maturity and sustain programs, policies, and activities over time. To benefit fully from the substantial investment in public health research and subsequent program development, we need to better understand what factors can promote long-term program sustainability (3).

The concept of program sustainability is not new, and theoretical work has been done in many fields, including business, health care administration, social services, and public health (4). Current conceptual reviews of sustainability document the breadth of the area and the lack of consensus around the definition for sustainability or sustainability determinants (5–7). A small number of integrated sustainability frameworks have been introduced, and these are important for guiding future research on program sustainability (4,8,9).

Although much conceptual work has been done on defining sustainability, a dearth of tools is available to scientists, evaluators, and public health program managers to assess sustainability. Of all the frameworks for sustainability, few have been translated into valid and reliable tools for measuring sustainability. In an extensive literature search, Hutchinson found references to 33 tools measuring some aspect of sustainability (5). However, only 4 of these tools had psychometric analyses available. In our own work, we found 17 frameworks for sustainability in the public health literature and only 2 tools for measuring sustainability. None of the tools had been tested for reliability or validity. Reliable and valid tools that are relevant for public health are needed to measure sustainability at the programmatic level.

We present a new assessment tool based on our program sustainability framework (4) that allows individual programs to assess their capacity for sustainability across 8 sustainability domains. This Program Sustainability Assessment Tool (PSAT) has been designed to assess sustainability for a wide variety of public health programs. The tool has been developed and tested on a large number of public health programs at both the community and state level. With the development of a reliable tool for measuring program sustainability capacity, individual programs will be able to conduct more efficient program planning and improvement. In addition, dissemination and implementation scientists will be better able to study how evidence-based programs can be sustained in real-world settings over time.

## Methods

This measurement development study was designed to produce a reliable assessment instrument that could be easily used to measure capacity for program sustainability. The structure of the PSAT was based on our previous sustainability conceptual framework (4). The design of the instrument was guided by 4 basic design principles: 1) short and easy to use; 2) usable by small and large programs (especially community and state-level programs); 3) applicable for a wide variety of program types in public health, and relevant for clinical and social service programs; and 4) useful as a scientific, evaluation, and program planning tool.

### Initial instrument development

On the basis of our prior literature review and concept mapping study (4), we developed a pilot version of the PSAT. Concept mapping is a type of structured conceptualization that can be used by groups to develop a conceptual framework to guide evaluation or planning (10). The pilot PSAT had 63 items and 9 sustainability domain subscales: Political Support [now called Environmental Support] (5 items), Funding Stability (7 items), Partnerships (9 items), Organizational Capacity (11 items), Program Evaluation (5 items), Program Adaptation (7 items), Communications (7 items), Public Health Impacts (6 items), and Strategic Planning (6 items). Each item assessed an element that was found to be related to sustainability by the literature review and concept mapping processes. Respondents assessed the degree to which each element was present in their program by using a Likert scale with anchors of 1 (“Little or no extent”) to 7 (“A very great extent”).

The pilot instrument development study was not designed as an in-depth validation study. However, we collected a small number of items from a sample of the participants that could be used for simple validation analyses. The focus of the PSAT is to accurately characterize the sustainability capacity of a public health program. As such, if the instrument is valid, the sustainability scores should reflect characteristics of the program, agency, or organization. Conversely, sustainability scores should not be strongly related to characteristics of the individual program directors or staff members who are filling out the PSAT.

### Data collection

We employed 2 primary tools to collect data: paper surveys administered in person at trainings and online surveys administered using Qualtrics (Qualtrics, Provo, Utah). The paper and electronic versions of the surveys were identical. Each was composed of 63 elements organized into the 9 original domains.

### Participating programs and respondents

The sustainability project advisory group identified initiatives (comprising multiple grantees with separate programs) and individual programs for a pilot test of the instrument (Table 1). To ensure a diverse sample of participants, we selected programs from 4 different chronic disease areas and 2 program sizes (state and community). Respondents from 252 programs completed the initial PSAT during trainings and evaluations from October 2010 through October 2011. From these sources, a data set was assembled to test the initial framework (n = 592; 386 completed all portions of the survey, 206 missed 1 item or more). Of the 592 surveys, 494 were completed online, and 98 used the paper version. The Washington University in St Louis and Saint Louis University institutional review boards approved research on this secondary data set. Participants per program ranged from 1 to 15, and program topics were tobacco control (n = 291 [49.2%]), obesity prevention (n = 221 [37.3%]), diabetes (n = 42 [7.1%]), and oral health (n = 35 [5.9%]). Three programs (0.5%) reported covering multiple areas. Of the participants, 375 (63.3%) focused their efforts at the state level, 212 (35.8%) focused at the community level, and 5 (0.8%) focused at the national level.

**Table 1:** Characteristics of Participating Programs in Tests of the Program Sustainability Assessment Tool

Participating Initiatives and Programs	Program Level	Program Focus	No. of Programs	No. of Participants
Missouri Healthy and Active Communities grantees	Community	Obesity Prevention	47	99
Appalachia Diabetes Coalitions	Community	Diabetes	31	31
Missouri Tobacco Prevention and Cessation Initiative grantees	Community	Tobacco	31	82
Centers for Disease Control and Prevention (CDC), Division of Nutrition, Physical Activity, and Obesity grantees	State	Obesity Prevention	50	114
Missouri Council for Activity and Nutrition Coalition	State	Obesity Prevention	1	8
CDC, Fall Institute workshop grantees	State	Diabetes, tobacco	21	24
Missouri Tobacco Control Program	State	Tobacco	1	11
CDC, Office on Smoking and Health grantees	State	Tobacco	53	142
CDC, Office on Smoking and Health Sustaining States grantees	State	Tobacco	4	46
CDC, Division of Oral Health grantees	State	Oral Health	13	35
**Total**			**252**	**592**

### Covariates

Several variables that described the respondents’ programs were used as covariates in the validation measure. These variables were program level, program type, role in program, years in program, and perceived sustainability. Program level had 2 options, state or community. Program type described the public health issue that the program focused on: obesity prevention, diabetes, oral health, tobacco, or multiple health topics. Role in program indicated the respondent’s role in their organization: manager/director, evaluator, financial/operations/business manager, program staff, board member, community partner, or other. “Years in program” was a continuous variable that indicated how long the respondent had been with the organization. Perceived sustainability was measured on a 7-point Likert scale with anchors of 1 (not at all sustainable) and 7 (very sustainable) in response to the question “How would you rate the overall sustainability of your program?” The perceived sustainability question was added partway through the pilot study, and 205 participants were able to respond to this question. Although we were limited in the types of covariate questions we could include in this pilot study, these 4 were included to capture potential differences in program sustainability capacity. For example, whether a program is organized at the state or community level may influence its access to resources or partnerships that support sustainability capacity.

### Analyses

Using the lavaan structural equation modeling package version 0.4-11 in the R statistical software (R Foundation for Statistical Computing, Vienna, Austria) (11), we conducted confirmatory factor analysis and basic psychometric analyses. Confirmatory factor analysis is a powerful and appropriate tool for testing a hypothesized subscale structure in a measurement instrument (12,13). Initially confirmatory factor analysis was applied to our entire data set to identify poorly performing items and test our hypothesized sustainability domain structure. Poor items were those that had low variability or poor fit with the intended subscale. Once the final structure was determined, we performed multiple-group confirmatory factor analysis to test for factorial invariance across levels of 2 covariates: program level and program type (14). For both the overall test and the multiple group tests, we used 3 measures of model fit to assess model adequacy: the comparative fit index (CFI), root mean square error of approximation (RMSEA), and the standardized root mean square residual (SRMR) (15).

## Results

### Instrument improvement and domain structure

The item and confirmatory factor analyses resulted in the final structure of the PSAT. Table 2 shows the improved psychometrics during the instrument development process. The baseline model (Table 2), which assumes no subscale structure, was used as a comparison for the pilot and final models. The pilot model included all of the initial 63 items contained in 9 subscales. After psychometric analyses, 23 items and 1 subscale (Public Health Impacts) were dropped from the pilot version of the framework and tool. Items were dropped if they had lower loadings in the latent factors, had poor variance (ie, restricted range), or had excessive missing data. The Public Health Impacts subscale was dropped because of high subscale intercorrelation and because we determined that Public Health Impacts measured a sustainability *outcome* rather than a program’s *capacity* for sustainability. The final PSAT comprised 40 items organized within 8 subscale domains. Each domain had 5 items. This simple and balanced structure facilitates training and scoring with programs and groups.

**Table 2:** Confirmatory Factor Analysis Results of Baseline, Pilot, and Final Program Sustainability Assessment Tool Instruments

Phase	Subscales	Items	χ^2^/*df*	CFI	RMSEA	SRMR	AIC
Baseline	1	63	15.3	0.58	0.102	0.087	114,884
Pilot	9	63	3.7	0.82	0.067	0.063	108,194
Final	8	40	3.6	0.89	0.066	0.055	69,518

Abbreviations: CFI, comparative fit index; RMSEA, root mean square error of approximation; SRMR, standardized root mean residual; AIC, Akaike Information Criterion.

Table 2 also shows the good fit of the 8-domain confirmatory factor analysis model to the data — that is, the 40-item PSAT did a credible job of measuring 8 important sustainability domains that were identified in previous work. The final 8-domain model has the lowest AIC of the three models, indicating better fit to the data. Although the CFI could be larger, the RMSEA of .066 was between good (.05) and acceptable (.08), and an SRMR smaller than 0.08 indicates good fit (16,17). Table 3 shows the final 40 items in the 8 subscales of the PSAT, along with the individual item-factor loadings (under the Total Sample column).

**Table 3:** Item-Factor Loadings for Final Itemized Subscales of the Program Sustainability Assessment Tool (PSAT) and Confirmatory Factor Analysis for Program Level and Program Type for Programs Participating in Tests of the PSAT

Subscale Definition and Items	Total Sample (n = 592)	Confirmatory Factor Analysis			
		Program Level		Program Type	
		Community (n = 212)	State (n = 380)	Tobacco (n = 301)	Nontobacco (n = 291)
**PoliticalSupport^a^: Internal and external political environments that support your program**					
1. Political champions advocate for the program.	0.84	0.84	0.84	0.82	0.84
2. The program has strong champions with the ability to garner resources.	0.81	0.74	0.82	0.82	0.80
3. The program has political support within the larger organization.	0.72	0.77	0.68	0.69	0.75
4. The program has political support from outside of the organization.	0.84	0.87	0.82	0.81	0.86
5. The program has strong advocacy support.	0.74	0.68	0.75	0.66	0.82
**Funding Stability: Establishing a consistent financial base for your program**					
6. The program exists in a supportive state economic climate.	0.61	0.44	0.66	0.57	0.66
7. The program implements policies to help ensure sustained funding.	0.66	0.66	0.64	0.64	0.68
8. The program is funded through a variety of sources.	0.61	0.59	0.63	0.62	0.60
9. The program has a combination of stable and flexible funding.	0.77	0.72	0.80	0.80	0.75
10. The program has sustained funding.	0.75	0.79	0.73	0.74	0.76
**Partnerships: Cultivating connections between your program and its stakeholders**					
11. Diverse community organizations are invested in the success of the program.	0.77	0.76	0.77	0.77	0.77
12. The program communicates with community leaders.	0.85	0.82	0.86	0.86	0.85
13. Community leaders are involved with the program.	0.85	0.81	0.88	0.86	0.85
14. Community members are passionately committed to the program.	0.78	0.74	0.78	0.78	0.78
15. The community is engaged in the development of program goals.	0.78	0.77	0.78	0.77	0.79
**Organizational Capacity: Having the internal support and resources needed to effectively manage your program**					
16. The program is well integrated into the operations of the organization.	0.77	0.68	0.80	0.75	0.79
17. Organizational systems are in place to support the various program needs.	0.84	0.73	0.86	0.85	0.83
18. Leadership effectively articulates the vision of the program to external partners.	0.81	0.78	0.78	0.84	0.79
19. Leadership efficiently manages staff and other resources.	0.84	0.85	0.80	0.85	0.85
20. The program has adequate staff to complete the program’s goals.	0.60	0.60	0.52	0.65	0.54
**Program Evaluation: Assessing your program to inform planning and document results**					
21. The program has the capacity for quality program evaluation.	0.78	0.74	0.78	0.78	0.78
22. The program reports short-term and intermediate outcomes.	0.82	0.78	0.83	0.82	0.81
23. Evaluation results inform program planning and implementation.	0.89	0.90	0.89	0.93	0.86
24. Program evaluation results are used to demonstrate successes to funders and other key stakeholders.	0.84	0.84	0.84	0.86	0.83
25. The program provides strong evidence to the public that the program works.	0.80	0.72	0.80	0.76	0.82
**Program Adaptation: Taking actions that adapt your program to ensure its ongoing effectiveness**					
26. The program periodically reviews the evidence base.	0.78	0.78	0.81	0.81	0.74
27. The program adapts strategies as needed.	0.89	0.87	0.89	0.92	0.85
28. The program adapts to new science.	0.86	0.84	0.88	0.90	0.81
29. The program proactively adapts to changes in the environment.	0.89	0.89	0.88	0.90	0.88
30. The program makes decisions about which components are ineffective and should not continue.	0.75	0.73	0.74	0.77	0.74
**Communications: Strategic communication with stakeholders and the public about your program**					
31. The program has communication strategies to secure and maintain public support.	0.89	0.89	0.88	0.91	0.88
32. Program staff members communicate the need for the program to the public.	0.86	0.85	0.84	0.85	0.88
33. The program is marketed in a way that generates interest.	0.85	0.81	0.83	0.86	0.84
34. The program increases community awareness of the issue.	0.83	0.74	0.81	0.85	0.81
35. The program demonstrates its value to the public.	0.81	0.62	0.82	0.85	0.77
**Strategic Planning: Using processes that guide your program’s directions, goals, and strategies**					
36. The program plans for future resource needs.	0.81	0.80	0.82	0.82	0.80
37. The program has a long-term financial plan.	0.83	0.85	0.84	0.80	0.84
38. The program has a sustainability plan.	0.82	0.83	0.81	0.77	0.84
39. The program’s goals are understood by all stakeholders.	0.74	0.64	0.74	0.82	0.67
40. The program clearly outlines roles and responsibilities for all stakeholders.	0.78	0.65	0.79	0.85	0.71

a This domain is now called Environmental Support.

As a further test of the PSAT structure, we conducted multiple-group confirmatory factor analysis to test for factorial invariance across levels of 2 covariates: program level and program type. This allowed us to determine whether the subscale structure of the PSAT is the same for different types of programs. These analyses tested the hypothesis that there were equal item-factor loadings across different subgroups. The results indicated a significant difference between community and state programs (χ^2^ = 78.0, degrees of freedom [*df*] = 32, *P* < .001), but no difference was detected between tobacco and other types of public health programs, including diabetes, obesity prevention, and oral health (χ^2^ = 46.0, *df* = 32, *P* = .052). For each of these tests, only 2 groups could be compared because of the small sample size in some of the subgroups. Although the results suggested that the item-factor structure is not identical between community and state programs, examination of the individual item loadings for the multiple group tests showed that the factor loadings are quite similar (see the right 4 columns in Table 3). The average item-loading difference between the program level groups was 0.05, and only 2 of the 40 items showed differences as large as 0.20. The large sample size for the confirmatory factor analysis may have led to detecting small differences in factor loading as significantly different. However, the pattern of the loadings for both program type and program level suggested that the PSAT has similar (if not identical) structure across different sorts of public health programs.

### Subscale reliability

The subscale reliabilities (internal consistency) for the PSAT were excellent, especially given the small size of each subscale (5 items) (18) (Table 4). The average internal consistency of the 8 subscales was 0.88 and ranged from 0.79 to 0.92. Furthermore, the item loadings showed consistently high correlations with their respective subscales, although the Funding Stability subscale had lower item loadings (Table 3).

**Table 4:** Subscale Reliabilities (Internal Consistency) for the Program Sustainability Assessment Tool

Subscale	Cronbach’s α
Political Support^a^	0.88
Funding Stability	0.79
Partnerships	0.90
Organizational Capacity	0.87
Program Evaluation	0.90
Program Adaptation	0.91
Communications	0.92
Strategic Planning	0.88

a This domain is now called Environmental Support.

### Preliminary PSAT results and validation

**Figure 1:**
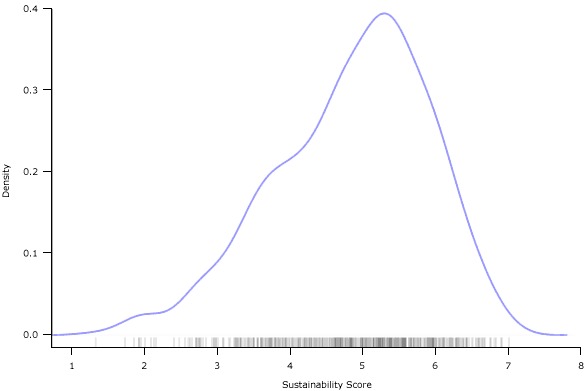
Density plot (frequency) of the variability of Program Sustainability Assessment Tool (PSAT) scores across 252 public health programs participating in tests of the PSAT.

Using the data obtained from the 592 participants of the PSAT pilot development, we created the final PSAT scale to be used for a small number of exploratory descriptive and validation analyses. The average total sustainability score across the 252 programs was 4.84, with a range of 1.32 to 7.00 and an interquartile range of 4.17 to 5.58. Scores had good coverage across the range of possible scores, although program participants were not likely to report extremely low sustainability scores (Figure 1).

**Figure 2:**
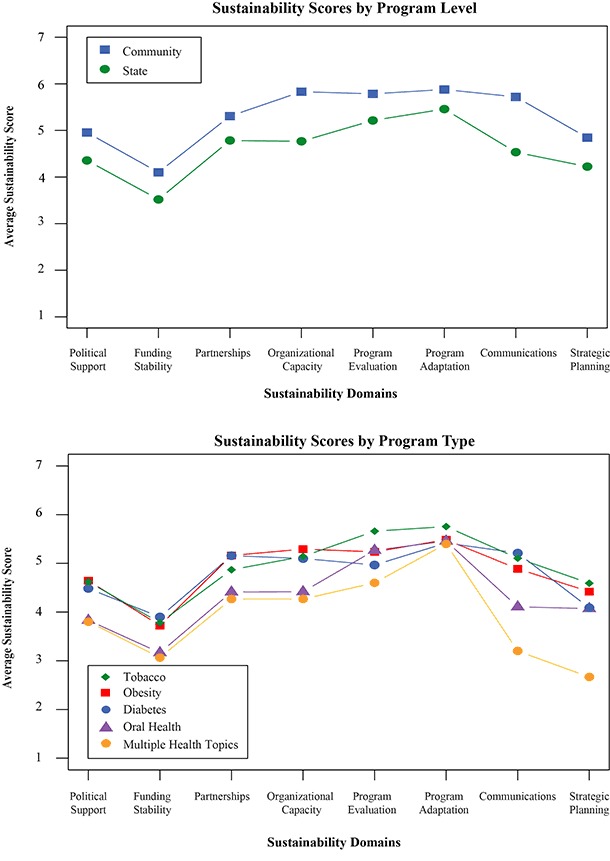
Program Sustainability Assessment Tool domain scores by level of program and type of program among programs participating in tests of the tool.

Analyses looking at the relationship between the overall sustainability scores and a small set of organizational and individual-level covariates showed that the sustainability scores obtained from the PSAT are significantly related to 2 important organizational predictors: type of program (F_4,587_ = 3.33, *P* = .01), and level of program (F_1,590_ = 70.6, *P* < .01). At the same time, PSAT scores are unrelated to 2 individual-level predictors: years in program (β = −0.005, not significant), and role in program (F_3,589_ = 0.09, not significant). This provides some discriminant validation evidence that the PSAT instrument is working as intended. The subscale scores vary by level and type of program (Figure 2). These preliminary analyses suggested that the PSAT is able to distinguish among different levels of sustainability that may be driven by program characteristics such as community or state level or focus of program.

Finally, we conducted a simple construct validity analysis. A sample of 205 of the pilot participants were asked, after filling out the PSAT and before receiving their results, to indicate their perception of their program’s general sustainability. If the PSAT were a valid measure of program sustainability, we would expect PSAT sustainability scores to be positively related to what program managers and staff perceive is the sustainability of the program. The correlations between the perceived sustainability scores and the PSAT overall sustainability scores as well as the 8 domain scores were as follows: overall = 0.68, Political Support = 0.48, Funding Stability = 0.67, Partnerships = 0.44, Organizational Capacity = 0.58, Program Evaluation = 0.45, Program Adaptation = 0.32, Communications = 0.55, Strategic Planning = 0.63. The formal PSAT sustainability scores had a moderate positive correlation with perceived program sustainability. The Funding Stability and Strategic Planning domains were most closely associated with general perceived program sustainability.

## Discussion

The results of our psychometric study of the PSAT indicated that the tool is reliable and ready to use for assessing a program’s capacity for sustainability by researchers, evaluators, and program managers and staff. The confirmatory factor analyses show that even with only 40 items, the PSAT is able to capture the distinct elements of program sustainability suggested by previous conceptual work (4). Each of the 8 subscales works well — the high internal consistency scores are notable, given the small size of each scale (5 items). The pilot data suggest that there is not a problem with restriction of range, because sustainability scores at the individual and program levels vary across most of the intended range of the instrument. This study contributes to dissemination and implementation science by exploring the factors involved in maintaining public health programs once they are implemented.

The PSAT was designed to be easy to use by a wide variety of public health and other social service programs. It is short, has a consistent structure that facilitates training, and can be used for numerous purposes including program monitoring, program evaluation, and strategic planning. A companion article in *Preventing Chronic Disease* provides more in-depth description of how the PSAT can be used for community and public health programs (19).

There are numerous research and evaluation next steps for the PSAT. A strength of this study is the diversity of public health program types used to test its psychometrics. However, despite this diversity, the tool has been used only with chronic disease programs. On the basis of confirmatory factor analysis, the poorly performing domain of Public Health Impacts was dropped. By removing this public health–specific domain, the tool can now be used by social service and clinical care programs as well. In particular, the promise of sustainability assessment for large-scale health systems is intriguing (20). Meanwhile, a slightly adapted version of the PSAT that modifies the Political Support domain to cover Environmental Support was introduced in late 2013. Future research and evaluation work needs to be done to ascertain the utility of the PSAT for different fields and types of interventions (21).

A second area of future work is to further validate the PSAT. Although the simple validation data presented here suggest that the sustainability scores obtained with the PSAT are associated with important organizational and program characteristics, this finding needs to be explored in more detail. For example, our data showed that across more than 250 programs, state programs have lower sustainability scores than community programs. Future work can tell us if this is a typical result and help to uncover the underlying causes. It is not surprising that state and local programs may differ in their sustainability. State programs are typically larger, exist in more diverse political environments, have broader partnership structures, and have typically been in existence longer than local programs, which tend to have shorter life cycles. More research is needed to identify the sustainability differences across program types.

The ultimate validation challenge for the PSAT is to use it to predict and understand long-term sustainability outcomes for public health and other types of programs (3). Program sustainability is an essential goal for public health if the promise of our investment in evidence-based programming and policies is to be realized. The PSAT is a reliable assessment tool that can help us make progress toward understanding what factors allow programs to sustain their effects over time.
